# Early life environmental antibiotic exposure and preschool allergic diseases: A biomonitoring-based prospective study in eastern China

**DOI:** 10.3389/fpubh.2022.1043942

**Published:** 2022-10-31

**Authors:** Hang Zheng, Xinxin Zeng, Qiuling Xie, Yuhang Wu, Quanhua Liu, Qian Chen, Lisu Huang, Weixi Zhang

**Affiliations:** ^1^Department of Pediatric Allergy and Immunology, The Second Affiliated Hospital and Yuying Children's Hospital of Wenzhou Medical University, Wenzhou, China; ^2^Department of Infectious Diseases, Xinhua Children's Hospital, Xinhua Hospital, Shanghai Jiao Tong University School of Medicine, Shanghai, China; ^3^MOE-Shanghai Key Laboratory of Children's Environmental Health, Xinhua Hospital, Shanghai Jiao Tong University School of Medicine, Shanghai, China; ^4^Department of Infectious Diseases, the Children's Hospital, Zhejiang University School of Medicine, National Clinical Research Center for Child Health, Hangzhou, China; ^5^Department of Pediatric Respiratory Diseases, Xinhua Children's Hospital, Xinhua Hospital, Shanghai Jiao Tong University School of Medicine, Shanghai, China

**Keywords:** allergy, asthma, environmental antibiotic, enrofloxacin, azithromycin, skin-prick-test

## Abstract

**Background:**

Globally, the prevalence of allergic diseases remains high, as does the level of environmental antibiotics. It has been found that clinical antibiotic application may increase preschool allergy risk. However, few biomonitoring studies have been conducted about the association between early life environmental trace dose antibiotic exposure and preschool allergy.

**Objective:**

To analyze the association between prenatal environmental antibiotic levels and allergic diseases using logistic regression models.

**Methods:**

A total of 743 pregnant women and their offspring from the Shanghai Allergy Birth Cohort completed five years follow-up, and 251 mother-infant pairs were finally included. Maternal urine samples were collected for 15 antibiotic quantitative measurements using liquid chromatography-tandem mass spectrometry. The high-antibiotic group was defined as having at least half of antibiotics exceeding the median concentration. Allergic diseases were assessed by clinicians through clinical history, standardized questionnaires, and annual physical examinations until the age of five. Skin-prick-test (SPT) was performed at 5 years old.

**Results:**

The incidence of allergic diseases was generally higher in the high-antibiotic than that in the low-antibiotic group. Compared to the low-comprehensive antibiotic group, children in the high-antibiotic group were weakly associated with allergic diseases but had a 6-fold increased risk of food allergens sensitivity (OR: 7.09, 95% CI: 1.59, 31.74). Association of above-median single prenatal antibiotic concentration exposure and allergic diseases was also observed (azithromycin and asthma, OR: 2.72, 95% CI: 1.15, 6.42; enrofloxacin and wheeze, OR: 2.22, 95% CI: 1.22, 4.05; trimethoprim and atopic dermatitis, OR: 2.00, 95% CI: 1.08, 3.71). Moreover, children with higher prenatal norfloxacin levels were more sensitive to food allergens (OR: 5.52, 95%CI: 1.54, 19.71).

**Conclusion:**

Early-life environmental antibiotic exposure may be correlated with an increased risk of asthma, wheeze, atopic dermatitis, and SPT positivity for food allergens in 5-year-old children.

## Introduction

Preferred-as-veterinary antibiotics (PVA) and veterinary antibiotics (VA), like sulfamethoxazole, trimethoprim, ofloxacin, and ciprofloxacin, are widely used in animal husbandry to prevent diseases (1). Because of this, environmental antibiotics have been posed as emerging pollution. It has been estimated that in 2030, the global consumption of human antibiotics (HA) might be 2-times higher than that in 2015, and the global consumption of PVA and VA will increase by 67% compared to that in 2010 (2, 3). Notably, residual antibiotics in the environment have been frequently detected in the surface and ground waters for daily drinking (4) and human food supply, including livestock and poultry products (meat, milk, and eggs), aquatic products, and vegetables (5, 6). Previous studies indicated that the fetus *in utero* is extensively exposed to environmental antibiotics as antibiotic residues are detected in almost half of maternal urine and neonatal meconium samples (7–9). These long-term residual environmental antibiotics may enter the maternal body through food and drinking water and cross the placenta during pregnancy, thus adversely affecting the immune health of the fetus, as experimental studies have indicated (10).

At the same time, the prevalence of allergic diseases has remained continuously high in recent years. Approximately one-third of children worldwide develop allergies, inevitably leading to global economic burdens (11, 12). The pathogenesis of allergic diseases may be a combination of environmental and immune genetic factors (13). It has been found that short-term high-dose clinical antibiotic use during pregnancy, assessed by electronic records or questionnaires, may increase asthma or wheeze risk in preschool offspring (14–17). In recent years, the introduction of liquid chromatography-mass spectrometry has allowed for the objective measurement and biological monitoring of antibiotic exposure (18). However, few studies have explored the association between environmental antibiotics exposure with quantitative detection and allergic diseases. A recent biomonitoring epidemiological study found that maternal exposure to certain VA or PVA during pregnancy was linked to a higher risk of asthma and eczema in 4-year-old children (19). However, their study lacked a biological explanation and information about food allergy. In contrast, our study looks further at the relationship between IgE and skin-prick-test for food allergens and common inhaled allergens with early-life antibiotic exposure, adding powerful biomarkers that could predict subsequent allergic disease development, including food allergy. Therefore, this study biomonitoring environmental antibiotic exposure from pregnant women's biological samples (in the third trimester) to validate the following hypotheses: (1) an association exists between early life environmental antibiotic exposure and allergy at 5 years old, and (2) a difference exists between high- and low-antibiotic-exposure groups in terms of allergy development by categorizing them according to detected antibiotic levels.

## Materials and methods

### Study population

The Shanghai Allergy Birth Cohort is an ongoing prospective birth cohort that recruited pregnant women from June 2012 from two tertiary-level hospitals in Shanghai, Xinhua Hospital and the International Peace Maternity and Child Health Hospital. Third-trimester pregnant women (29–41 gestational weeks) who underwent routine prenatal examinations at the two hospitals were considered for this cohort. Eventually, 1,143 mother-infant pairs participated in the cohort as a baseline, and 743 pairs (65%) completed each scheduled follow-up until their children were 5 years old. A total of 198 pairs were followed up only by telephone and did not fill out the standard questionnaire in the field and were therefore excluded. Moreover, 294 pairs whose biological samples were missing; hence, 251 mother-infant pairs were finally included in the study (Supplementary Figure S1). Most of the 251 pregnant women reported no use of antibiotics during pregnancy, there might have been unreported cases.

Participants completed standard questionnaires upon enrollment and at 6 months, 1 year, 2 years, and 5 years of the child's age. The initial questionnaire collected information on maternal age, weight, parental education levels, and parental atopic-disease history (i.e., atopic dermatitis, allergic rhinitis, or asthma). The subsequent questionnaires at 6 months, 1 year, 2 years, and 5 years of the age focused on the children's fundamental conditions and allergic diseases (including cough and wheeze) and covered the International Study of Asthma and Allergies in Childhood (ISAAC) questionnaire. All participants signed an informed consent form at enrollment. The study was approved by the Ethics Committees of the involved research institutions and hospitals.

### Maternal Urinary antibiotic concentrations

In this study, maternal urine samples collected 1–3 days before delivery were assessed for antibiotic exposure. According to previous studies regarding environmental antibiotic exposure (4, 5, 7, 20), this study detected 15 antibiotics from five classes, including four fluoroquinolones (norfloxacin, enrofloxacin, ciprofloxacin, and ofloxacin), four sulfonamides (sulfamethazine, sulfamethoxazole, sulfamethoxazole, and trimethoprim), three phenicols (thiamphenicol, florfenicol, and chloramphenicol), three macrolides (clarithromycin, erythromycin, and azithromycin), and tetracycline. Clarithromycin and erythromycin were excluded from the subsequent analysis for non-detection and sulfamethazine and chloramphenicol were excluded from the further analysis for low detection frequency (below 10%). Among the 13 antibiotics, two were VAs, two HAs, and nine PVAs.

Specimens were stored at Xinhua Hospital's biobank at −80°C. The urine samples were preprocessed (21), and liquid chromatography–tandem mass spectrometry was subsequently employed to analyze the 15 antibiotics' urinary concentrations according to the methods previously described (8). Dr. Ehrenstorfer (Augsburg, Germany) and Toronto Research Chemicals (Toronto, ON, Canada) provided isotope-labeling standards (sulfamethoxazole-d_4_, sulfadiazine-^13^C_6_, ciprofloxacin-d_8_, enrofloxacin-d_5_, trimethoprim-d_3_, chloramphenicol-d_5_, azithromycin-d_3_, and tetracycline-d_6_). Quality control samples were randomly included among samples in each batch and the purity of all the standards was strictly maintained at ≥ 94%. The limit of detection (LOD) was set at 0.1–1.0 ng/mL for the selected antibiotics, with a signal-to-noise ratio of 3.

Urinary creatinine levels were detected using the enzymatic method (Roche Cobas 8000 chemistry analyzer, Roche, Basel, Switzerland). The creatinine-corrected urinary antibiotic concentrations (ng/g creatinine) were considered the internal exposure levels, which were calculated using volume-based urinary antibiotic concentrations divided by urinary creatinine concentrations.

### Diagnosis of asthma, wheeze, atopic dermatitis, and diarrhea

Asthma was diagnosed by professional pediatric respiratory physicians at 1, 2, 3, 4, and 5 years of age *via* repeated symptoms (wheeze and cough), history of allergy to dust mites, and clinical examination (immunoglobulin E [IgE] level and pulmonary function tests), based on the Global Initiative for Asthma guidelines (22). Wheeze was characterized by a continuous whistling sound occurring during breathing, suggesting narrowing or obstruction in some respiratory-airway parts (23). According to the criteria of Williams et al. (24), atopic dermatitis was diagnosed *via* the symptom of an itchy rash and at least three of the following characteristics: (1) history of flexural involvement; (2) onset under 2 years old; (3) medical history of asthma or allergic rhinitis; (4) ever had dry skin; and (5) visible flexural dermatitis. Parents reported information on diarrhea during the second year after birth. The definition of diarrhea depended on the change of stool characteristics and frequency: the formation of loose stools, watery stools, egg-patterned stools, or stools with mucus or pus and blood; the number of stools is more than usual or more than three times.

### Definition of skin prick test (SPT)-related outcomes

Skin prick test (SPT) was conducted for nine common inhalants and nine food allergens, including dust mites (*Dermatophagoides pteronyssinus* and *Dermatophagoides farina*), cat/dog dander, *Aspergillus niger*, ragweed/indus/birch tree/willow pollen, egg, cow's milk, mango, sea shrimp, sea crab, beef, mutton, cashew nut, and walnut. In this study, SPT for common inhalants and nine food allergens were performed by pediatricians at 5 years old according to the European standardization SPT procedures, with histamine and physiologic saline as positive and negative controls, respectively. A positive result was confirmed after 15–20 min of application when the wheal diameter was at least 3 mm larger than that of the negative control (25). Moreover, one or more positive results for the nine common inhalants or nine food allergens were considered as SPT positivity for statistical analysis.

### Statistical analysis

Mean values (standard deviations [SDs]), medians (P_25_, P_75_), and frequency (percentage, %) were calculated for sociodemographic characteristics, antibiotic concentrations in maternal urine samples, and allergy-related health outcomes among group participants. Furthermore, corresponding-antibiotic-category detection in maternal urine samples was defined as the overall antibiotic detection in that category covered in this study. Multivariate logistic regression was used to analyze the associations between antibiotic exposure *in utero* and allergy-related health outcomes. Three analyses were mainly conducted: (1) based on the concentrations and detection frequencies of 13 antibiotics, the total population was classified into binaries, and children in the high-antibiotic group were regarded as having higher comprehensive prenatal environmental antibiotic exposure. A globally accepted definition of high-antibiotic exposure is not available currently. Thus, the high-antibiotic group was defined as (1) having at least half of antibiotics exceeding the median out of eight antibiotics whose detection frequency exceeded 25%; (2) for five single antibiotics whose frequency was above 50%, antibiotic concentrations were divided into binaries by their medians. Antibiotic exposure concentrations (≥ median) were regarded as having higher single prenatal environmental antibiotic exposure; (3) concerning six single antibiotics whose frequency below 50%, the population was spilled into binaries (concentrations: < 90th percentile and ≥ 90th percentile). Antibiotic exposure concentrations (≥ 90th percentile) were considered as higher single environmental antibiotic exposure *in utero*. The results are presented as adjusted odd ratios (ORs) and 95% confidence intervals (CIs). The following potential confounders were selected: offspring sex, maternal education status at delivery, gestational age, breastfeeding during the first 6 months, and offspring body mass index (BMI) at 5 years of age. Data were missing in 0.7–2.1% of the mother-infant pairs for one or more potential confounders. Additionally, regarding SPT positivity for common inhaled allergens or food allergens, 10.4–17.9% of the participants lacked corresponding data. Despite partial data loss in some participants, they were included in this study's analysis.

All analyses were performed using Empower R (www. empowerstats.com, X&Y solutions, Inc., Boston MA, USA) and R software (http://www.T-project.org). *P* ≤ 0.05 was considered statistically significant.

## Results

### Baseline characteristics

Table 1 shows the baseline maternal and infant characteristics. The pregnant women's mean age was 29.29 years, and they predominantly held Bachelor's degrees (91.63%). Almost all neonates were full-term (98.41%), and approximately half were male. All children had Apgar scores > 7 at birth. The average BMI at age 5 years was 15.27 kg/m^2^. Moreover, 27.49% of the offspring received breastmilk during the first 6 months.

**Table 1:** Basic characteristics and children reported allergy-related outcomes of mother-infant pairs stratified by antibiotic concentration in the Shanghai Allergy Birth Cohort (*N* = 251).

**Characteristics**	**Mean** ±**SD / median (P**_**25**_**-P**_**75**_**) /** ***n*** **(%)**			***P-*value**
	**Overall (*N* = 251)**	**Low-antibiotic group (*n* = 113)**	**High-antibiotic group (*n* = 138)**	
**Maternal characteristics**				
Maternal age, years	29.29 ± 3.34	29.49 ± 3.48	29.12 ± 3.22	0.387
Maternal pre-pregnancy body mass index, kg/m^2^	21.58 ± 3.22	21.99 ± 3.55	21.25 ± 2.90	0.071
Maternal education				0.762
<Bachelor	21 (8.37)	9 (7.96)	12 (8.70)	
Bachelor	207 (82.47)	92 (81.42)	115 (83.33)	
>Bachelor	23 (9.16)	12 (10.62)	11 (7.97)	
Han ethnic	241 (96.02)	108 (95.58)	133 (96.38)	0.452
Positive maternal allergy	34 (13.55)	13 (11.50)	21 (15.22)	0.409
Gestational age (weeks)				0.840
Preterm birth (≤ 36 weeks)	4 (1.59)	2 (1.77)	2 (1.45)	
Full-term birth (37–41 weeks)	247 (98.41)	111 (98.23)	136 (98.55)	
Multiple parity	18 (7.17)	10 (8.85)	8 (5.80)	0.351
Mode of delivery				0.950
Natural labor	208 (83.20)	93 (83.04)	115 (83.33)	
Cesarean	42 (16.80)	19 (16.96)	23 (16.67)	
**Child characteristics**				
Male	123 (52.56)	51 (49.51)	72 (54.96)	0.407
Breastfeeding (6 months)	69 (27.49)	27 (23.89)	42 (30.43)	
Birth weight, kg	3.44 ± 0.42	3.45 ± 0.39	3.43 ± 0.45	0.729
Apgar score, >7	251 (100)	113 (100)	137 (99.28)	0.365
Weight at 5 years of age, kg	19.02 ± 2.99	18.94 ± 3.16	19.08 ± 2.86	0.729
Height at 5 years of age, cm	110.02 ± 4.20	109.88 ± 3.90	110.12 ± 4.43	0.656
Body mass index at 5 years of age, kg/m^2^	15.27 ± 1.30	15.62 ± 1.88	15.67 ± 1.60	0.833
Frequency of respiratory tract infections before 5 years of age	14 (8, 19)	13 (7, 16)	13 (8, 21)	0.061
IgE concentration at 5 years of age (ng/mL)	54.75 (22.32, 206.13)	55.66 (24.37, 212,20)	54.73 (21.17, 198.60)	0.510
IgG concentration at 5 years of age (ng/mL)	9.00 (7.00, 10.00)	9.00 (7.00, 10.00)	8.00 (7.00, 9.00)	0.361

Ig, immunoglobulin; SPT, skin prick test.

### Environmental antibiotic concentrations in maternal urine

Table 2 shows that the total antibiotic-detection frequency in pregnant women reached 98.01%. The detection frequencies of the 13 single antibiotics varied from 1.20% (sulfamethazine) to 80.48% (norfloxacin). The median of total urinary creatinine-corrected antibiotic concentration was 702.84 ng/g creatinine, and VA and PVA concentrations were 25.07 ng/g and 579.11 ng/g creatinine, respectively. It implicated PVA as the dominant antibiotic in maternal urine samples. Moreover, fluoroquinolones exhibited the highest concentration (263.52 ng/g creatinine) among the five classes of antibiotics that the study completely detected. Regarding single-antibiotic comparisons, ciprofloxacin exhibited the highest median urinary creatinine-corrected concentration (78.29 ng/g creatinine), followed by norfloxacin (43.74 ng/g creatinine).

**Table 2:** Urinary creatinine-corrected concentrations (ng/g creatinine) of 13 antibiotics.

**Antibiotic**	**Usage**	**N > LOD (%)**	**Median (P**_**25**_ **-P**_**75**_ **)**		
			**Overall**	**Low–antibiotic group**	**High–antibiotic group**
**All antibiotics** ^**a**^		246 (98.01)	702.84 (402.67–1325.29)	392.55 (271.66–642.66)	1099.18 (688.83–1895.72)
HAs		44 (17.53)	–	–	–
VAs		194 (77.29)	25.07 (16.48–40.53)	17.19 (<LOD−22.60)	34.02 (24.97–57.11)
PVAs		245 (97.61)	579.11 (310.87–1165.48)	291.69 (227.51–491.58)	879.69 (569.49–1672.49)
VAs+PVAs		246 (98.01)	611.86 (341.43–1255.52)	316.29 (240.08–554.06)	934.43 (603.44–1785.29)
**Sulfonamides** ^**b**^		217 (86.45)	73.79 (45.40–117.92)	45.59 (30.46–67.11)	102.31 (69.77–181.84)
Sulfamethazine^**d**^	PVA	3 (1.20)	–	–	–
Sulfamethoxazole	PVA	171 (68.13)	26.58 (<LOD−46.29)	19.49 (<LOD−31.13)	34.39 (<LOD−55.67)
Sulfadiazine	PVA	97 (38.65)	<LOD (<LOD−17.47)	<LOD (<LOD−9.36)	<LOD (<LOD−22.61)
Trimethoprim^c^	PVA	140 (55.78)	14.63 (<LOD−39.04)	<LOD (<LOD−12.67)	27.90 (<LOD−64.89)
**Fluoroquinolones** ^**b**^		238 (94.82)	263.52 (149.68–445.30)	149.66 (104.67–239.49)	347.50 (245.75–710.89)
Norfloxacin	PVA	202 (80.48)	43.74 (16.31–125.98)	26.82 (14.51–73.28)	66.66 (22.61–165.85)
Enrofloxacin	VA	181 (72.11)	15.38 (<LOD−24.11)	9.62 (<LOD−13.62)	20.06 (15.32–33.33)
Ciprofloxacin	PVA	157 (62.55)	78.29 (<LOD−156.29)	42.63 (<LOD−69.04)	120.57 (<LOD−207.76)
Ofloxacin	PVA	108 (43.03)	<LOD (<LOD−99.82)	<LOD (<LOD−47.84)	78.58 (<LOD−139.06)
**Phenicols** ^**b**^		92 (36.65)	<LOD (<LOD−65.78)	<LOD (<LOD−41.79)	<LOD (<LOD −86.80)
Thiamphenicol	PVA	42 (16.73)	–	–	–
Florfenicol	VA	52 (20.72)	–	–	–
Chloramphenicol^**d**^	HA	18 (7.17)	–	–	–
**Macrolides** ^**b**^					
Azithromycin	HA	28 (11.16)	–	–	–
**Tetracyclines** ^**b**^					
Tetracycline	PVA	77 (30.68)	<LOD (<LOD−367.25)	–	<LOD (<LOD−553.31)

LOD, limit of detection; HA, human antibiotic; PVA, preferred–as–veterinary antibiotic; VA, veterinary antibiotic.

a Total concentration of all antibiotics;

b Total concentration of antibiotics in the corresponding antibiotic category;

c Because trimethoprim is usually administered in combination with sulfonamides, it was analyzed together with sulfonamides;

d Sulfamethazine and chloramphenicol were excluded from the subsequent analysis for the detection below 10%. –; No value due to below LOD.

The high-antibiotic group was defined as having at least half of the antibiotics exceeding the median. The median serum concentrations of total IgE at the age of 5 in the high- and low-antibiotic groups were 55.66 ng/mL and 54.73 ng/mL, respectively. Further, the baseline characteristics of mother-infant pairs between the high- and low-antibiotic groups were not significantly different (all *P* > 0.05) (Table 1).

### Prenatal environmental antibiotic exposure and allergy-related outcomes

Of the 251 mother-infant pairs, the incidence of allergy-related outcomes in the high-antibiotic group was higher than that in the low-antibiotic group, except for diarrhea (asthma: 22.46 vs. 18.58%; wheeze: 27.54 vs. 19.47%; atopic dermatitis: 23.19 vs. 20.54%; SPT positivity for common inhaled allergens: 47.11 vs. 43.88%; SPT positivity for food allergens: 14.16 vs. 2.27%; diarrhea: 43.27 vs. 44.16%). However, no statistically significant differences in the incidence of allergy-related outcomes were observed between the low-antibiotic group and the high-antibiotic group, except for SPT being sensitive to food allergens (SPT positivity for food allergens: 14.16 and 2.27%, *P* < 0.05) (Table 3). The risk of SPT positivity for food allergens in the high-antibiotic group was 6.09 times higher (OR: 7.09, 95% CI: 1.59, 31.74) than that in the low-antibiotic group. This elevated association decreased after adjusting for potential confounders; nevertheless, it remained evident (OR: 6.03, 95% CI: 1.21, 30.04). There was no statistically significant association between asthma, wheeze, atopic dermatitis, and SPT positivity for common inhaled allergens and high-exposure antibiotics in the unadjusted and adjusted models (Table 3). Moreover, another grouping method was applied to distinguish the high- and low-antibiotic groups, and the association between the two groups with allergic diseases was similar to that in the above-mentioned analysis (Supplementary Table S1).

**Table 3:** The associations of high maternal urinary antibiotic concentrations with allergic diseases, skin prick test positivity for food allergens and common inhaled allergens at age 5 years, and diarrhea at age 2 years.

**Allergy–related outcomes**	***n* (%)^a^**	**Crude odds ratio**	**Adjusted odds ratio**
		**(95% CI)**	**(95% CI)**
**Wheeze**	60 (23.90)		
Low–antibiotic group	22 (19.47)	Ref	Ref
High–antibiotic group	38 (27.54)	1.57 (0.87, 2.86)	1.20 (0.60, 2.42)
**Asthma**	52 (20.72)		
Low–antibiotic group	21 (18.58)	Ref	Ref
High–antibiotic group	31 (22.46)	1.27 (0.68, 2.36)	1.05 (0.51, 2.17)
**Atopic dermatitis**	55 (21.91)		
Low–antibiotic group	23 (20.54)	Ref	Ref
High–antibiotic group	32 (23.19)	1.17 (0.64, 2.14)	1.10 (0.55, 2.17)
**Diarrhea**	79 (43.65)		
Low–antibiotic group	34 (44.16)	Ref	Ref
High–antibiotic group	45 (43.27)	0.96 (0.53, 1.75)	0.86 (0.43, 1.72)
**SPT positivity for common inhaled allergen**s	100 (45.45)		
Low–antibiotic group	43 (43.88)	Ref	Ref
High–antibiotic group	57 (47.11)	1.14 (0.67, 1.95)	0.90 (0.49, 1.66)
**SPT positivity for food allergens**	18 (8.96)		
Low–antibiotic group	2 (2.27)	Ref	Ref
High–antibiotic group	16 (14.16)^*^	**7.09 (1.59, 31.74)**	**6.03 (1.21, 30.04)**

CI, confidence interval; SPT, skin prick test.

a The number and percentage of allergic diseases, diarrhea, and SPT positivity for food allergens and common inhaled allergens in the low–antibiotic group and the high–antibiotic group.

* The symbol indicates significant differences between the incidence of the low–antibiotic group and the high–antibiotic group.

Models for adjusted odd ratios include offspring sex, maternal education status at delivery, gestational age, breastfeeding during the first 6 months, and offspring body mass index at 5 years of age.

In the analysis of each class of antibiotics, VA exposure above the median showed an 84% higher risk (OR: 1.84, 95% CI: 1.02, 3.34) of wheeze than lower concentration exposure; there was no significant association between the allergic diseases and the concentrations of other categories of antibiotics (Supplementary Table S2).

Regarding five specific antibiotics whose frequency was above 50%, compared to those with low-antibiotic exposure, children with exposure to above-median prenatal enrofloxacin (i.e., ≥ 15.38 ng/g creatinine) and trimethoprim (i.e., ≥ 14.63 ng/g creatinine) concentrations appeared 2.22-times (OR: 2.22, 95% CI: 1.22, 4.05) and 2.00-times (OR: 2.00, 95% CI: 1.08, 3.71) more likely to develop wheeze and atopic dermatitis, respectively. Furthermore, the risk of SPT positivity for food allergens in children with norfloxacin exposure concentrations above the medians (i.e., ≥ 43.74 ng/g creatinine) increased 4.52 times (OR: 5.52, 95% CI: 1.54, 19.71) compared with children with lower antibiotic exposure concentrations (Table 4). A higher antibiotic concentration (≥ medians) did not increase the risk of SPT positivity for common inhaled allergens in 5-year-olds and diarrhea in 2-year-olds (Supplementary Tables S3, S4).

**Table 4:** Associations between maternal urinary antibiotic concentrations (ng/g creatinine) and allergic diseases and skin prick test positivity for food allergens (five antibiotics with detection frequency above 50%).

**Antibiotic**	**Asthma**	**Wheeze**	**Atopic dermatitis**	**SPT positivity for food allergens**
	**Crude odds ratio (95% CI)**			
**Sulfonamides**				
**Sulfamethoxazole**				
<Median (26.58)	Ref	Ref	Ref	Ref
≥Median (26.58)	0.99 (0.54, 1.82)	0.91 (0.51, 1.62)	0.60 (0.32, 1.09)	1.51 (0.57, 3.99)
**Trimethoprim**				
<Median (14.63)	Ref	Ref	Ref	Ref
≥Median (14.63)	0.90 (0.49, 1.65)	0.91 (0.51, 1.62)	**2.00 (1.08, 3.71)**	2.07 (0.74, 5.74)
**Fluoroquinolones**				
**Norfloxacin**				
<Median (43.74)	Ref	Ref	Ref	Ref
≥Median (43.74)	0.99 (0.54, 1.82)	1.18 (0.66, 2.11)	1.24 (0.68, 2.26)	**5.52 (1.54, 19.71)**
**Enrofloxacin**				
<Median (15.38)	Ref	Ref	Ref	Ref
≥Median (15.38)	1.79 (0.96, 3.34)	**2.22 (1.22, 4.05)**	1.13 (0.62, 2.05)	2.02 (0.73, 5.62)
**Ciprofloxacin**				
<Median (78.29)	Ref	Ref	Ref	Ref
≥Median (78.29)	1.20 (0.65, 2.22)	1.54 (0.86, 2.77)	0.71 (0.39, 1.29)	1.59 (0.59, 4.28)

CI, confidence interval; SPT, skin prick test.

We further analyzed the six single antibiotics whose frequency was below 50% by grouping the population into two sets (< 90th percentile and ≥ 90th percentile) (Table 5). Among the six antibiotics analyzed, only azithromycin was positively associated with asthma (OR: 2.72, 95% CI: 1.15, 6.42). No relationships were found between the other five antibiotics and related allergic outcomes.

**Table 5:** Associations between maternal urinary antibiotic concentrations (ng/g creatinine) and allergic diseases and skin prick test positivity for food allergens (seven antibiotics with detection frequency below 50%).

**Antibiotic**	**Asthma**	**Wheeze**	**Atopic dermatitis**	**SPT positivity for food allergens**
	**Crude odds ratio (95% CI)**			
**Sulfonamides**				
**Sulfadiazine**				
<90th percentile (29.05)	Ref	Ref	Ref	Ref
≥90th percentile (29.05)	1.47 (0.58, 3.72)	1.48 (0.61, 3.60)	1.35 (0.54, 3.40)	1.22 (0.26, 5.76)
**Fluoroquinolones**				
**Ofloxacin**				
<90th percentile (175.82)	Ref	Ref	Ref	Ref
≥90th percentile (175.82)	0.90 (0.32, 2.52)	0.95 (0.36, 2.49)	1.07 (0.41, 2.81)	0.48 (0.06, 3.80)
**Phenicols**				
**Thiamphenicol**				
<90th percentile (37.06)	Ref	Ref	Ref	Ref
≥90th percentile (37.06)	0.67 (0.22, 2.04)	0.74 (0.26, 2.04)	1.07 (0.41, 2.81)	0.51 (0.06, 4.03)
**Florfenicol**				
<90th percentile (28.01)	Ref	Ref	Ref	Ref
≥90th percentile (28.01)	0.90 (0.32, 2.52)	1.20 (0.48, 3.00)	0.62 (0.20, 1.87)	0.43 (0.05, 3.39)
**Macrolides**				
**Azithromycin**				
<90th percentile (52.75)	Ref	Ref	Ref	Ref
≥90th percentile (52.75)	**2.72 (1.15, 6.42)**	2.19 (0.93, 5.12)	1.35 (0.54, 3.40)	0.57 (0.07, 4.59)
**Tetracyclines**				
**Tetracycline**				
<90th percentile (1054.96)	Ref	Ref	Ref	Ref
≥90th percentile (1054.96)	0.47 (0.14, 1.63)	0.38 (0.11, 1.33)	1.07 (0.41, 2.81)	2.79 (0.83, 9.43)

CI, confidence interval; SPT, skin prick test.

### IgE levels between the SPT-negative and the SPT-positive groups

Moreover, SPT-positive individuals were found to have considerably greater IgE levels than negative individuals among children whose mothers had lower concentrations of antibiotics found in their urine. When exposed to higher single antibiotic levels (including trimethoprim, norfloxacin, enrofloxacin, ciprofloxacin, thiamphenicol, and azithromycin), children who tested positive for SPT had noticeably higher IgE levels than those who tested negative for SPT (Supplementary Table S5).

## Discussion

This study assessed the associations between environmental antibiotic exposure *in utero* and allergy-related health outcomes in preschool children until the age of 5 years and found that compared to the comprehensive antibiotic low-exposure group, children with higher comprehensive prenatal environmental antibiotic exposure were not associated with allergic diseases but may be more liable to test positive for SPT for food allergens. Children exposed to higher environmental enrofloxacin and trimethoprim *in utero* were correlated with an increased risk of wheeze and atopic dermatitis, respectively. Children with higher prenatal norfloxacin levels were more sensitive to food allergens than lower-concentration exposure. Furthermore, prenatal environmental azithromycin exposure was related to the susceptibility to asthma compared to children with lower exposure.

Compared with older children and adults, developing fetuses are more susceptible to adverse effects from environmental compounds with possible lifelong changes in respiratory and immune health (26). In our previous study, the fetus was exposed to multiple environmental antibiotics *in utero*, which may have resulted in exposure to doses heavier than their body weight can withstand and may adversely affect children's health (27). To date, the association between short-term high-dose clinical antibiotic application and allergic diseases has been investigated, but few studies have explored the association between long-term low-dose environmental antibiotics exposure and allergies. Previous studies have illuminated that prenatal clinical antibiotic use may be positively associated with asthma, wheeze, and atopic dermatitis in preschool children (14–17). A recent epidemiological biomonitoring study found associations between prenatal low-dose antibiotic exposure and current asthma and eczema in 4-year-old children (19). In our study, long-term environmental antibiotic exposure *in utero* was correlated with an increased risk of asthma, wheeze, and atopic dermatitis in 5-year-old children, consistent with the previous studies, especially the studies related to the short-term high-dose clinical antibiotic application. Furthermore, as can be determined, SPT in 2-year-old had a clinically useful ability to predict asthma and atopic dermatitis in subsequent years (28). However, no correlation was observed between prenatal environmental trace dose antibiotic exposure and SPT positivity for common inhaled allergens in 5-year-old in our study, which delineated that prenatal environmental trace dose antibiotic exposure has no effect on allergic diseases after the age of 5.

Regarding food allergy, no biomonitoring study has yet looked into the connection between environmental antibiotics exposure and food allergy. Nevertheless, some research evaluated the association between clinical antibiotic exposure by employing questionnaires and electronic medical records and food allergy. A meta-analysis (including three correlated studies) (17) summarized no associations between prenatal clinical antibiotic exposure and food allergy in the offspring. Notably, contrary to the previous research studies regarding clinical antibiotic use, our results showed that low-dose environmental antibiotics exposure might increase the positive SPT rate for food allergens in offspring, which is one of the reliable methods to screen for food allergies (29). In addition, our study found that SPT-positive individuals have considerably greater IgE levels at the age of 5 than negative individuals among children with lower or high single antibiotics concentrations exposure, which is one of the vital indicators for evaluating food allergy. However, it makes no significant statistical difference between the high- and low-antibiotic groups in diarrhea incidence at the age of 2, which are additional diagnostic tools for food allergy. Collectively, it may indicate that long-term low-dose environmental antibiotic exposure in early life has a more adverse impact on preschool children to develop food allergy than short-term high-dose antibiotic exposure. Hence, our study adds evidence to the associations between prenatal exposure to low-dose environmental antibiotics and offspring allergies. It contributes to understanding the potential adverse effects of cumulative prenatal exposure to low-dose antibiotics on fetal allergies and emphasizes the need to regulate the residual concentrations of such antibiotics in the environment.

The mechanism underlying environmental antibiotic exposure *in utero* leading to increased allergic disease risk remains unclear. There is a potential explanation that environmental antibiotic exposure could damage the immune defense and inflammatory response, impacting the gut microbiome and metabolic pathways, thereby mediating allergic diseases, which are closely related to immune defense and inflammation. Several experimental studies demonstrated that antibiotic exposure would eventually threaten human health by disrupting the balance maintained by the microbiota and damaging immune defense (10, 30, 31). An experimental study in zebrafish found that early exposure to environmental levels of sulfamethoxazole delayed the hatchment, reduced the body length, weakened the host immune defense to pathogens, and finally triggered immune and inflammatory response of healthy zebrafish larvae, with Toll-like receptors playing a significant role in regulating this immune response (10). Noticeably, our previous research demonstrated that long-term exposure to environmental concentrations of sulfamethoxazole and oxytetracycline potentially altered the composition and abundance of zebrafish gut microbiome, including the higher abundance of pathogenic Flavobacterial species. The alternation of relative abundance of metabolic pathways for fatty acid biosynthesis and amino acids was also observed (30). In mice with food allergy compared to control groups, our group also discovered changes in the diversity, evenness, and richness of the bacterial community together with Trp metabolism, which are involved with Firmicutes and Akkermansia (32). To some extent, our findings support the results of immunotoxicity in these experimental studies. Nonetheless, these findings need to be verified through further biological mechanism research.

Our study has several methodological strengths, including the prospective cohort base and 5-year follow-up period. The study's data collection and valid outcome assessment were prospective and conducted by professionals, thus precluding retrospective bias. Adopting a quantitative detection of antibiotic concentration to describe antibiotic exposure and detecting an abundant variety of antibiotics were additional features. Additionally, none of the 251 pregnant women reported using any antibiotics analyzed in the study during pregnancy, excluding the effect of iatrogenic antibiotic exposure on allergy-related outcomes.

This study has certain limitations. First, antibiotic exposure was assessed based on maternal urine samples collected in the third trimester. However, previous studies demonstrated that environmental antibiotics' detection frequency and urinary concentration differed little in the first, second, and third pregnancy trimesters (7). In addition, environmental antibiotics exposure is generally correlated to behavioral and dietary habits, and it would not be prone to change in the short term. Therefore, the urine samples and detection in the study are considered stable to reflect early life environmental antibiotic exposure. Second, food allergy is a kind of allergic disease, but it was not directly evaluated in this study. The diagnosis of food allergy depends on the clinical history to determine whether the symptoms are related to allergy (including vomit and diarrhea) and tests, including SPT and oral food challenge (29). The oral food challenge may trigger severe allergic reactions. Hence, our study assessed clinical history and SPT positivity for food allergens, which was sensitive and reliable for the diagnosis of food allergy and precluded the missed diagnosis. Third, breastfeeding is associated with subsequent allergy diseases in children (33), but the breastfeeding rate was quite low compared to other countries. However, breastfeeding rates did not differ statistically among different antibiotic-level groups in our analysis, and the association of environmental antibiotics with allergic disease outcomes remained with only slight changes after the adjustment of breastfeeding. Finally, clinical antibiotic use in children may act as a mediating factor between perinatal environmental antibiotics and allergic disease in 5-year-old children because environmental antibiotics may affect gut microbiota and immune function, leading to increased infectious diseases and clinical antibiotics use, resulting in childhood allergic diseases. However, assessing the 5-year application of clinical antibiotics is quite complex, with variable types and doses of antibiotics, as well as periods of use and intermittent periods. In future studies, we will attempt to investigate more deeply the interaction of clinical and environmental antibiotics on allergic diseases in children.

## Conclusions

In our population-based analysis, preschool children are widely exposed to multiple environmental antibiotics in Shanghai. In addition, prenatal exposure to certain environmental antibiotics (azithromycin, enrofloxacin, trimethoprim, and norfloxacin) was associated with an increased risk of subsequent asthma, wheeze, atopic dermatitis, and positive results of SPT to food allergens in preschool children. In this respect, more evidence is needed to clarify the association and the specific pathway it affects.

## Data availability statement

The raw data supporting the conclusions of this article will be made available by the authors, without undue reservation.

## Ethics statement

The studies involving human participants were reviewed and approved by Xinhua Hospital and International Peace Maternity and Infant Health Hospital Affiliated with the Shanghai Jiao Tong University School of Medicine. Written informed consent to participate in this study was provided by the participants' legal guardian/next of kin.

## Author contributions

HZ and XZ contributed equally for formal analysis, roles and writing—original draft, and writing—review editing. QX, YW, QL, and QC were responsible for data curation. WZ and LH contributed equally to project administration and supervision. All authors contributed to the article and approved the submitted version.

## Funding

This research was supported by the National Natural Science Foundation of China (grant numbers 81874265 and 82073561), Shanghai Science and Technology Commission (grant numbers 18411966600 and 19410740800), Shanghai Jiao Tong University School of Medicine (grant number 2020002), Key Discipline Construction Plan from Shanghai Municipal Health Commission (GWV-10.1-XK01), and National Key Specialty of Children's Respiration.

## Conflict of interest

The authors declare that the research was conducted in the absence of any commercial or financial relationships that could be construed as a potential conflict of interest.

## Publisher's note

All claims expressed in this article are solely those of the authors and do not necessarily represent those of their affiliated organizations, or those of the publisher, the editors and the reviewers. Any product that may be evaluated in this article, or claim that may be made by its manufacturer, is not guaranteed or endorsed by the publisher.
