# Supplementary material for: Early life environmental antibiotic exposure and preschool allergic diseases: A biomonitoring-based prospective study in eastern China
Source: Front Public Health. 2022 Oct 31;10:1043942. doi: 10.3389/fpubh.2022.1043942 (PMC9659984; doi:10.3389/fpubh.2022.1043942)
Supplement: Supplementary file 1 [file Data_Sheet_1.docx]

**Supporting Information**

**Table of Contents**

**Use of logistic regression analysis to assess the effects of prenatal antibiotic exposure on allergy-related outcomes (asthma, wheeze, atopic dermatitis, diarrhea caused by food allergy, and SPT positivity for food allergens and common inhaled allergens) in offspring. Additionally, we divided the respiratory infections into three groups according to frequency and subsequently analyzed the association between respiratory infections and allergic diseases using logistic regression analysis. We performed linear-trend tests by entering the median value of each respiratory-infection category as a continuous variable.**

**Table S1.** The associations of high maternal urinary antibiotic concentrations with allergic diseases, skin prick test (SPT) positivity for food allergens and common inhaled allergens at age 5 years, and diarrhea at age 2 years.

**Table S2.** Associations between maternal urinary antibiotic concentrations (ng/g creatinine) in the category and allergic diseases and skin prick test positivity for food allergens (N=251)

**Table S3.** Associations between maternal urinary antibiotic concentrations (ng/g creatinine) and skin prick test positivity for common inhaled allergens and diarrhea (five antibiotics with detection frequency above 50%)

**Table S4.** Associations between maternal urinary antibiotic concentrations (ng/g creatinine) and skin prick test positivity for common inhaled allergens and diarrhea (seven antibiotics with detection frequency below 50%)

**Table S5.** IgE levels associations between the SPT-negative and the SPT-positive groups in the low or high antibiotic populations

**Figure S1.** Flow chart showing a selection of cohort participants

Table S1. The associations of high maternal urinary antibiotic concentrations with allergic diseases, skin prick test positivity for food allergens and common inhaled allergens at age 5 years, and diarrhea at age 2 years.

| Allergy-related outcomes | n (%)^a^ | Crude odds ratio  (95% CI) | Adjusted odds ratio  (95% CI) |
| --- | --- | --- | --- |
| **Wheeze** | 60 (23.90) |  |  |
| Low-antibiotic group | 35 (23.33) | ref | ref |
| High-antibiotic group | 25 (24.75) | 1.01 (0.54, 1.88) | 0.84 (0.41, 1.71) |
| **Asthma** | 52 (20.72) |  |  |
| Low-antibiotic group | 31 (20.67) | ref | ref |
| High-antibiotic group | 21 (20.79) | 1.08 (0.60, 1.95) | 0.84 (0.40, 1.77) |
| **Atopic dermatitis** | 55(21.91) |  |  |
| Low-antibiotic group | 32 (21.48) | ref | ref |
| High-antibiotic group | 23 (22.77) | 1.08 (0.59, 1.98) | 1.02 (0.51, 2.05) |
| **Diarrhea** | 79 (43.65) |  |  |
| Low-antibiotic group | 52 (49.06) | ref | ref |
| High-antibiotic group | 27 (36.00) | 0.58 (0.32, 1.07) | 0.67 (0.33, 1.35) |
| **SPT positivity for common inhaled allergen**s | 100 (45.45) |  |  |
| Low-antibiotic group | 61 (46.56) | ref | ref |
| High-antibiotic group | 39 (44.32) | 0.91 (0.53, 1.57) | 0.82 (0.44, 1.55) |
| **SPT positivity for food allergens** | 18 (8.96) |  |  |
| Low-antibiotic group | 5 (4.20) | ref | ref |
| High-antibiotic group | 13 (15.85) * | **4.30 (1.47, 12.57)** | **4.73 (1.28, 17.44)** |

Abbreviations: CI, confidence interval; SPT, skin prick test.

^a^ The number and percentage of allergic diseases, diarrhea, and SPT positivity for food allergens and common inhaled allergens in the low-antibiotic group and the high-antibiotic group.

* The symbol indicates significant differences between the incidence of the low-antibiotic group and the high-antibiotic group.

The high-antibiotic group was defined as an individual with at least five antibiotics above the median concentrations.

Models for adjusted odd ratios include offspring sex, maternal education status at delivery, gestational age, breastfeeding during the first 6 months, and offspring body mass index at 5 years of age.

Table S2. Associations between maternal urinary antibiotic concentrations (ng/g creatinine) in the category and allergic diseases and skin prick test positivity for food allergens (N=251)

| Antibiotic | Asthma | Wheeze | | Atopic dermatitis | SPT positivity for food allergens |
| --- | --- | --- | --- | --- | --- |
|  | | | Crude odds ratio (95% CI) | | |
| **All antibiotics^a^** |  |  | |  |  |
| <median (702.84) | ref | ref | | ref | ref |
| ≥median (702.84) | 0.61 (0.33, 1.13) | 0.58 (0.32, 1.05) | | 0.85 (0.47, 1.55) | 1.52 (0.56, 4.10) |
| **VAs** |  |  | |  |  |
| <median (25.07) | ref | ref | | ref | ref |
| ≥median (25.07) | 1.62 (0.87, 3.00) | **1.84 (1.02, 3.34)** | | 0.94 (0.51, 1.70) | 1.24 (0.47, 3.27) |
| **PVAs** |  |  | |  |  |
| <median (579.11) | ref | ref | | ref | ref |
| ≥median (579.11) | 0.82 (0.44, 1.50) | 0.76 (0.42, 1.36) | | 0.71 (0.39, 1.29) | 2.81 (0.96, 8.19) |
| **VAs+PVAs** |  |  | |  |  |
| <median (611.86) | ref | ref | | ref | ref |
| ≥median (611.86) | 0.82 (0.44, 1.50) | 0.76 (0.42, 1.36) | | 0.64 (0.35, 1.18) | 2.69 (0.92, 7.84) |
| **Sulfonamides^b^** |  |  | |  |  |
| <median (73.79) | ref | ref | | ref | ref |
| ≥median (73.79) | 1.20 (0.65, 2.22) | 1.29 (0.72, 2.31) | | 1.03 (0.56, 1.87) | 0.79 (0.30, 2.10) |
| **Fluoroquinolones^b^** |  |  | |  |  |
| <median (263.52) | ref | ref | | ref | ref |
| ≥median (263.52) | 0.99 (0.54, 1.82) | 1.08 (0.60, 1.93) | | 0.94 (0.51, 1.70) | 2.63 (0.90, 7.67) |

Abbreviations: CI: confidence interval; PVA, preferred-as-veterinary antibiotic; SPT, skin prick test; VA, veterinary antibiotic.

^a^ Total concentration of all antibiotics; ^b^ Total concentration of antibiotics in the corresponding antibiotic category.

Table S3. Associations between maternal urinary antibiotic concentrations (ng/g creatinine) and skin prick test positivity for common inhaled allergens and diarrhea (five antibiotics with detection frequency above 50%)

| Antibiotic | SPT positivity for common inhaled allergens | Diarrhea |
| --- | --- | --- |
|  | Crude odds ratio (95% CI) | |
| **Sulfonamides** |  |  |
| Sulfamethoxazole |  |  |
| <median (26.58) | ref | ref |
| ≥median (26.58) | 0.86 (0.48, 1.54) | 0.67 (0.39, 1.15) |
| Trimethoprim^c^ |  |  |
| <median (14.63) | ref | ref |
| ≥median (14.63) | 1.63 (0.90, 2.95) | 1.14 (0.67, 1.94) |
| **Fluoroquinolones** |  |  |
| Norfloxacin |  |  |
| <median (43.74) | ref | ref |
| ≥median (43.74) | 0.99 (0.55, 1.77) | 1.09 (0.64, 1.85) |
| Enrofloxacin |  |  |
| <median (15.38) | ref | ref |
| ≥median (15.38) | 0.80 (0.44, 1.44) | 1.02 (0.60, 1.73) |
| Ciprofloxacin |  |  |
| <median (78.29) | ref | ref |
| ≥median (78.29) | 0.80 (0.44, 1.44) | 0.63 (0.37, 1.08) |

Abbreviations: CI: confidence interval; SPT, skin prick test.

Table S4. Associations between maternal urinary antibiotic concentrations (ng/g creatinine) and skin prick test positivity for common inhaled allergens and diarrhea (seven antibiotics with detection frequency below 50%)

| Antibiotic | SPT positivity for common inhaled allergens | Diarrhea |
| --- | --- | --- |
|  | Crude odds ratio (95% CI) | |
| **Sulfonamides** |  |  |
| Sulfadiazine |  |  |
| <90th percentile (29.05) | ref | ref |
| ≥90th percentile (29.05) | 1.83 (0.75, 4.47) | 0.39 (0.14, 1.13) |
| **Fluoroquinolones** |  |  |
| Ofloxacin |  |  |
| <90th percentile (175.82) | ref | ref |
| ≥90th percentile (175.82) | 0.74 (0.31, 1.79) | 0.56 (0.19, 1.68) |
| **Phenicols** |  |  |
| Thiamphenicol |  |  |
| <90th percentile (37.06) | ref | ref |
| ≥90th percentile (37.06) | 0.36 (0.13, 1.04) | 0.80 (0.30, 2.18) |
| Florfenicol |  |  |
| <90th percentile (28.01) | ref | ref |
| ≥90th percentile (28.01) | 0.56 (0.23, 1.37) | 1.18 (0.46, 3.07) |
| **Macrolides** |  |  |
| Azithromycin |  |  |
| <90th percentile (52.75) | ref | ref |
| ≥90th percentile (52.75) | 0.60 (0.24, 1.49) | 0.73 (0.27, 1.95) |
| **Tetracyclines** |  |  |
| Tetracycline |  |  |
| <90th percentile (1054.96) | ref | ref |
| ≥90th percentile (1054.96) | 0.60 (0.24, 1.49) | 1.20 (0.48, 2.98) |

Abbreviations: CI, confidence interval; SPT, skin prick test.

Table S5. IgE levels associations between the SPT-negative and the SPT-positive groups in the low or high antibiotic populations

| IgE level (Mean+SD) | Low antibiotic concentration | | | High antibiotic concentration | | |
| --- | --- | --- | --- | --- | --- | --- |
|  | SPT-negative | SPT-positive | *P* value | SPT-negative | SPT-positive | *P* value |
| Sulfamethoxazole | 54.98 ± 95.44 | 340.41 ± 419.70 | <0.001* | 115.66 ± 349.67 | 221.61 ± 296.25 | 0.132 |
| Sulfadiazine | 83.27 ± 267.25 | 298.41 ± 386.64 | <0.001* | 128.62 ± 201.19 | 241.92 ± 308.79 | 0.369 |
| Trimethoprim | 93.20 ± 329.42 | 334.88 ± 356.93 | <0.001* | 79.16 ± 150.26 | 249.42 ± 390.82 | 0.006* |
| Norfloxacin | 115.77 ± 349.56 | 288.71 ± 382.94 | 0.022* | 54.86 ± 95.80 | 291.66 ± 372.47 | <0.001* |
| Enrofloxacin | 101.35 ± 340.39 | 283.07 ± 358.15 | 0.012* | 71.35 ± 140.27 | 297.12 ± 394.98 | <0.001* |
| Ciprofloxacin | 100.09 ± 358.09 | 301.53 ± 351.95 | 0.006* | 75.63 ± 141.30 | 276.15 ± 406.89 | <0.001* |
| Ofloxacin | 88.64 ± 275.74 | 294.75 ± 379.27 | <0.001* | 73.97 ± 142.29 | 244.12 ± 352.70 | 0.134 |
| Thiamphenicol | 88.91 ± 277.00 | 261.35 ± 327.83 | <0.001* | 74.36 ± 154.50 | 781.63 ± 747.47 | 0.002* |
| Florfenicol | 87.90 ± 275.59 | 293.54 ± 384.76 | <0.001* | 79.73 ± 158.81 | 244.22 ± 218.88 | 0.074 |
| Azithromycin | 93.72 ± 278.22 | 281.10 ± 374.94 | <0.001* | 34.26 ± 27.45 | 418.29 ± 391.73 | 0.003* |
| Tetracycline | 72.66 ± 262.92 | 284.18 ± 366.13 | <0.001* | 176.64 ± 247.80 | 375.27 ± 523.14 | 0.256 |

* The symbol indicates significant differences in IgE levels between the SPT-negative and the SPT-positive groups in the low or high antibiotic populations.

High or low antibiotic concentration is related to Table S3 and S4.


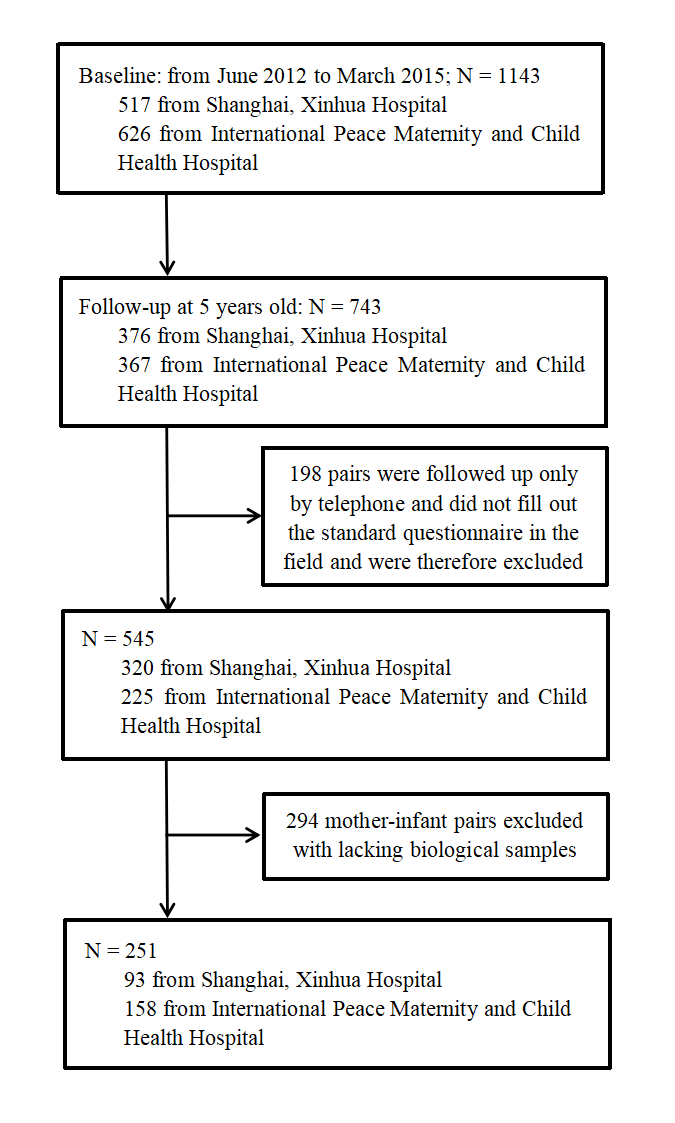


Figure S1. Flow chart showing a selection of cohort participants
